# On the Sensibility of Abdominal Organs and the Influence of Injections of Cocaine upon It

**Published:** 1907-04

**Authors:** 


### ON THE SENSIBILITY OF ABDOMINAL ORGANS AND THE INFLUENCE OF INJECTIONS OF COCAINE UPON IT.

---

L. Kast and S. J. Meltzer present a preliminary communication based on animal experimentation on the subject of abdominal pain. The prevailing view based upon exact surgical observations is that the abdominal organs, whether normal or inflamed, possess no sensation of pain. The writers, on the other hand, have found in animal experimentation that the sense of pain is present in normal organs, attacked. Pain along the supraorbital and infraorbital nerves, or deep behind the nose with a unilateral or bilateral nasal discharge of mucopus or pus, usually means that one or more of the accessory sinuses are involved.

The cardiac complications are bradycardia, tachycardia, irregular pulse, and attacks of syncope that may rapidly prove fatal. Malignant endocarditis due to the influenza bacillus may occur.

It is difficult to say positively that an isolated case of gastro-intestinal disturbance is due to the influenza poison. But when the disease is epidemic, the similarity in type of cases argues for a common cause. Six chief types are described.

1. Catarrh of the stomach, or of the intestines, or more frequently of both in the same attack.

2. Inflammation of a more severe type, affecting the same parts.

3. Cases which stimulate typhoid fever.

3. Cases of a choleraic type.

5. Cases which stimulate ulcerative colitis.

6. Cases of anomalous type.

The symptoms seem in large part to be due to the action of the toxins on the nerve cells and the nerve endings of controlling the various viscera. Several remarkable cases of irritation stimulating obstruction and paralysis with enormous abdominal distention are described.

There seems to be no room for doubt that there is some relation between influenza and appendicitis. Many observers have noted this relationship. Cases of appendicitis occurring in epidemic form coincidentally with influenza epidemics are on record. With a subsidence of the influenza there was a corresponding decrease in the number of cases of appendicitis.

The Neuroses and Psychoses that occur as complications or sequelae of influenza are of many types and of differing grades of and that it is considerably augmented in inflamed organs; and that a subcutaneous or intramuscular injection of cocaine is capable of completely abolishing this sensation in normal as well as in inflamed organs. They suggest that the anesthesia of the internal abdominal organs observed by certain surgeons was due to the use of cocaine. These investigators have also found that the injection of a small dose of cocaine has a calming influence upon the excitation of the narcotized and operated animals. These new observations are capable of practical application in medicine.—Medical Record, December 29, 1906.

